# Red Blood Cell Adenylate Energetics Is Related to Endothelial and Microvascular Function in Long COVID

**DOI:** 10.3390/biomedicines12030554

**Published:** 2024-03-01

**Authors:** Marzena Romanowska-Kocejko, Agata Jędrzejewska, Alicja Braczko, Klaudia Stawarska, Oliwia Król, Marika Frańczak, Gabriela Harasim, Ryszard T. Smoleński, Marcin Hellmann, Barbara Kutryb-Zając

**Affiliations:** 1 Department of Cardiac Diagnostics, Medical University of Gdansk, 80-210 Gdansk, Poland; marzena.romanowska-kocejko@gumed.edu.pl (M.R.-K.); marcin.hellmann@gumed.edu.pl (M.H.); 2 Department of Biochemistry, Medical University of Gdansk, 80-211 Gdansk, Poland; agata.jedrzejewska@gumed.edu.pl (A.J.); alicja.braczko@gumed.edu.pl (A.B.); klaudia.stawarska@gumed.edu.pl (K.S.); oliwia.krol@gumed.edu.pl (O.K.); marika.franczak@gumed.edu.pl (M.F.); gabriela.harasim@gumed.edu.pl (G.H.); rt.smolenski@gumed.edu.pl (R.T.S.)

**Keywords:** long COVID, red blood cells, nucleotides, microcirculation, endothelium

## Abstract

Adenine nucleotides play a critical role in maintaining essential functions of red blood cells (RBCs), including energy metabolism, redox status, shape fluctuations and RBC-dependent endothelial and microvascular functions. Recently, it has been shown that infection with the severe acute respiratory syndrome coronavirus-2 (SARS-CoV-2) might lead to morphological and metabolic alterations in erythrocytes in both mild and severe cases of coronavirus disease (COVID-19). However, little is known about the effects of COVID-19 on the nucleotide energetics of RBCs nor about the potential contribution of nucleotide metabolism to the long COVID syndrome. This study aimed to analyze the levels of adenine nucleotides in RBCs isolated from patients 12 weeks after mild SARS-CoV-2 infection who suffered from long COVID symptoms and to relate them with the endothelial and microvascular function parameters as well as the rate of peripheral tissue oxygen supply. Although the absolute quantities of adenine nucleotides in RBCs were rather slightly changed in long COVID individuals, many parameters related to the endothelial and microcirculatory function showed significant correlations with RBC adenosine triphosphate (ATP) and total adenine nucleotide (TAN) concentration. A particularly strong relationship was observed between ATP in RBCs and the serum ratio of arginine to asymmetric dimethylarginine—an indicator of endothelial function. Consistently, a positive correlation was also observed between the ATP/ADP ratio and diminished reactive hyperemic response in long COVID patients, assessed by the flow-mediated skin fluorescence (FMSF) technique, which reflected decreased vascular nitric oxide bioavailability. In addition, we have shown that patients after COVID-19 have significantly impaired ischemic response parameters (IR max and IR index), examined by FMSF, which revealed diminished residual bioavailability of oxygen in epidermal keratinocytes after brachial artery occlusion. These ischemic response parameters revealed a strong positive correlation with the RBC ATP/ADP ratio, confirming a key role of RBC bioenergetics in peripheral tissue oxygen supply. Taken together, the outcomes of this study indicate that dysregulation of metabolic processes in erythrocytes with the co-occurring endothelial and microvascular dysfunction is associated with diminished intracellular oxygen delivery, which may partly explain long COVID-specific symptoms such as physical impairment and fatigue.

## 1. Introduction

Infection with SARS-CoV-2 can lead to a range of temporary health problems, varying from mild to severe. According to NIH and WHO guidelines, mild COVID-19 is characterized in individuals who have any of the various signs and symptoms, e.g., fever, cough, sore throat, malaise, headache, muscle pain, nausea, vomiting, diarrhea, and loss of taste and smell [1]. It has been shown that many individuals after SARS-CoV-2 infection reported experiencing long-lasting COVID-19 sequelae and complications such as fatigue, dyspnea or chest pain. This condition, called long COVID, according to NICE (the National Institute for Clinical Excellence) guidelines, is commonly used to describe signs and symptoms that continue or develop after the acute phase of infection, including both ongoing symptomatic COVID-19 (from 4 to 12 weeks) and post-COVID-19 syndrome (PCS, 12 weeks or more) [2].

The mechanisms underlying PCS involve viral toxicity, immune dysregulation, hyperinflammation, hypercoagulability or endothelial damage. It has been also suggested that peripheral factors limiting O_2_ supply may explain the reduced cardiovascular fitness and muscular weakness, as persistently impaired systemic tissue oxygenation beyond an acute COVID-19 infection has been demonstrated. One potential reason for tissue hypoxemia in PCS might be associated with dysfunction in the microcirculation. It was shown that SARS-CoV-2 affects the microcirculation, causing endothelial cell swelling and damage (endotheliitis), microscopic blood clots (microthrombosis), capillary congestion, and damage to pericytes that are integral to capillary integrity and barrier function, tissue repair (angiogenesis) and scar formation [3].

On the other hand, decreased tissue perfusion in PCS may be linked to changes in oxygen uptake into the red blood cells (RBCs), oxygen binding or oxygen release. These occurrences may be connected to harm to the beta-chain of hemoglobin or elevated production of methemoglobin, leading to increased oxygen affinity in the unaffected hemoglobin [4]. During the active phase of the infection, there may be changes in the hematological profile, such as a decrease in RBC count or a shift in RBC distribution width. Additionally, alterations in the morphology, structure and function of RBCs could take place, offering an additional explanation for the symptoms described [5,6]. COVID-19 is also reported to enhance RBC deformability and aggregation, which can affect blood flow and reduce tissue oxygen supply [7]. Recently, it has been shown that not only does severe COVID-19 induce prominent RBC structural and rheological changes, but these effects can occur also in patients after a mild course of the disease. Impairment of RBC deformability, aggregated strength and morphological changes were shown to affect blood flow dynamics and, together with the left shifting of the oxygen dissociation curve, possibly oxygen supply in the microcirculation [8].

RBCs, unlike other cell types, cannot generate purine nucleotides through the de novo pathway. Instead, RBCs must engage the salvage reactions, recycling purine bases and nucleosides [9]. The synthesis of adenosine triphosphate (ATP) from adenosine diphosphate (ADP) in RBCs is exclusively dependent on the anaerobic conversion of glucose via the Embden–Meyerhof–Parnas pathway, as matured RBCs lack mitochondria [10]. The energy stored in ATP is crucial for various essential functions in erythrocytes, including oxygen delivery to the tissues, maintenance of the electrolyte gradient across erythrocyte membrane, synthesizing glutathione, preserving the asymmetry of the phospholipid membrane and keeping iron of hemoglobin (Hgb) in the ferrous state [10].

Multiple factors can impact the energy status of erythrocytes, leading to reductions in ATP concentration as well as in the adenosine triphosphate/adenosine diphosphate ratio (ATP/ADP) and adenylate energy charge (AEC). These factors include RBC enzymopathies [11], decreased erythrocyte deformability [12], a sedentary lifestyle [13], and neurodegenerative [14] and metabolic [15] disorders. However, very little is known about the metabolic alterations in erythrocytes after COVID-19. An individual study revealed significant changes in RBCs related to an increase in the glycolytic pathway to the detriment of the pentose phosphate pathway (PPP), highlighted by a characteristic increase in glucose consumption accompanied by an accumulation of intermediates of glycolysis and higher levels of phosphofructokinase (PFK), the rate-limiting enzyme of glycolysis [16]. Despite this knowledge, there is limited information available regarding the long-term influence of COVID-19 on the energy status of RBCs. This study aimed to analyze the levels of adenine nucleotides in RBCs isolated from patients, on average 12 weeks after mild SARS-CoV-2 infection, suffering from long COVID symptoms. RBC nucleotide levels were then related to the parameters of peripheral tissue oxygen supply as well as endothelial and microvascular function.

## 2. Materials and Methods

### 2.1. Participants

All participants in the study gave written consent following the principles of the Declaration of Helsinki. The study received approval from the Independent Bioethics Committee for Scientific Research at the Medical University of Gdansk, Poland (no. NKBBN/55/2021). The participants enrolled in the study consisted of cardiology outpatients exhibiting persistent symptoms associated with long COVID and healthy individuals with no previous diagnosis of SARS-CoV-2 infection (controls), as described in Table 1 and Table S1.

The diagnosis of COVID-19 was established through confirmation via polymerase chain reaction (PCR), serological testing or a rapid antigen test, meeting the sensitivity and specificity criteria recommended by the World Health Organization (WHO) for rapid antigen tests, within a maximum time frame of 4 months from the initial positive test result. The sample collection occurred before the vaccination period, specifically during February–March 2021. A specific time frame from the diagnosis to sample collection and flow-mediated skin fluorescence (FMSF) testing for each patient is provided in Table S1. None of the participants had received any vaccine doses during the study period. Detailed characteristics of the patients recruited for the study, including comorbidities and medications taken, are presented in Table 1 and Table S1.

### 2.2. Peripheral Blood Sampling and Morphology

Blood samples were collected into three separate tubes with ethylenediaminetetraacetic acid (EDTA) (2.7 mL), lithium heparinate (4.9 mL) as an anticoagulant and without any anticoagulant (S-monovette, Sarstedt, Nümbrecht, Germany). The first tube was used for the determination of peripheral blood morphology parameters using standard methods. The whole blood in the second tube was centrifuged (1000× *g*, 5 min, rt). The plasma and buffy coat were removed and the erythrocytes were washed three times with the buffered 0.9% sodium chloride (NaCl) solution and centrifuged each time (1000× *g*, 5 min, 4 °C). After a final wash, the resulting erythrocyte pellet was resuspended with a small volume of phosphate-buffered saline (PBS). Next, the samples of washed erythrocytes were deproteinized with an equal volume of 1.3 mol/L HClO_4_, mixed and then centrifuged at 16,000× *g* for 5 min at 4 °C. The supernatant (600 μL) was neutralized with 130–160 μL of 3 mol/L K_3_PO_4_ (to pH 5–7). The samples were centrifuged again under the same conditions as before, and the supernatant was immediately deep-frozen at −80 °C until the analysis of erythrocyte purine nucleotides (ATP, ADP and AMP). Whole blood in the third tube was centrifuged (5000 rpm, 5 min, rt) to obtain serum that was immediately frozen at −80 °C for later analyses.

### 2.3. Erythrocyte Nucleotide Measurements

The measurements were performed using ultra-high-performance liquid chromatography (UHPLC) with a UV–Vis detection system according to a previous method [17]. Briefly, 2 μL of supernatant was injected into a UHPLC system consisting of a Nexera LC40 set and an SPD-M30A diode array detector equipped with a high-sensitivity, 85 mm optical path cell (Shimadzu, Kyoto, Japan). Analytes were separated on a ReproSil-Pur 120 C18-AQ (150 × 2.0 mm ID, 4 µm) column using gradient elution at a flow rate of 500 µL/min. Peaks were detected by absorbance at 254 nm. After conversion to Hct, the intra-erythrocyte concentrations of purine nucleotides were expressed as μmol/L RBC. The values of ATP/ADP, ADP/AMP, total adenine nucleotide pool (TAN = ATP + ADP + AMP) and adenylate energy charge (AEC = [ATP] + 0.5 [ADP])/([ATP] + [ADP] + [AMP]) were later calculated.

### 2.4. Serum Amino Acid Measurements

Serum amino acid concentrations, including glycine, arginine, citrulline, asymmetric dimethylarginine (ADMA) and symmetric dimethylarginine (SDMA), were determined using liquid chromatography/mass spectrometry (LC/MS) as previously described [18]. Briefly, an aliquot of serum (50 µL) was enriched with internal standards and extracted using 100 µL of acetonitrile for 15 min on ice. Subsequently, the samples were centrifuged at 4 °C, 20,800× *g* for 10 min. The supernatants were collected and freeze-dried. The obtained sediments were dissolved in 100 µL of distilled water and analyzed by using ion-pair high-performance liquid chromatography with mass detection in positive mode electrospray ionization. To identify individual amino acids, their molecular weight, chromatographic retention time and fragmentation pattern were used, as described previously [19].

### 2.5. Serum hs-CRP Measurement

The concentration of serum high-sensitive C-reactive protein (hs-CRP) was measured using an Automated Photometer (ERBA XL-180, Mannheim, Germany) and specific ERBA kits according to the manufacturer’s instructions.

### 2.6. Microvascular Function Measurements

Microvascular function was evaluated in both female and male individuals who had recovered from COVID-19 (*n* = 19) and age/sex-matched controls (*n* = 5) without a history of COVID-19. The assessment utilized flow-mediated skin fluorescence (FMSF), a non-invasive optical technique that examines microcirculation and metabolic regulation by measuring NADH fluorescence intensity in the epidermis. The quantification of FMSF was carried out using AngioExpert, developed by Angionica Ltd. (Lodz, Poland), as previously outlined [20]. Upon reaching the microcirculation laboratory, participants were positioned within a temperature-regulated environment (24 ± 1 °C). Following a 15-min adjustment period, the baseline intensity of a reduced form of nicotinamide adenine dinucleotide (NADH) fluorescence was measured for 3 min on the forearm. Subsequently, blood flow within the brachial artery was temporarily halted for 3 min by applying pressure to a cuff positioned on the left upper arm, inflated to 50 mmHg above systolic blood pressure. Throughout the occlusion phase, NADH fluorescence was continuously monitored within the same region of the forearm. Upon cuff release, the reduction in NADH fluorescence was observed and recorded for 3 min.

Various parameters were recorded during NADH fluorescence measurement, including ischemic response (IR max; IR index) and hyperemic response (HR max; HR index). Direct measurements of oscillations in the reperfusion stage allowed for the evaluation of hypoxia sensitivity (HS), representing the intensity of flow motion associated with myogenic oscillations. The reactive hyperemia response (RHR) parameter, derived from the sum of IR max and HR max, reflected vascular endothelial function in connection with nitric oxide production during occlusion-induced hyperemia in blood vessels.

### 2.7. Statistical Analysis

The statistical analysis of the collected data was conducted utilizing InStat software (GraphPad Prism 9.0, San Diego, CA, USA). To assess normality, the Kolmogorov–Smirnov test, Shapiro–Wilk test, or D’Agostino and Pearson Omnibus test were employed. Group mean values were compared through unpaired Student’s *t*-test or Mann–Whitney test. Correlations were examined using Pearson correlation coefficient. The specific value of ‘n’ was provided for each experiment, and statistical significance was set at *p* < 0.05.

## 3. Results

### 3.1. Long COVID Patients Demonstrate Decreased Peripheral Tissue Oxygenation with Changes in Endothelial and Microvascular Function Parameters

In our study, we demonstrated lower levels of skin tissue oxygenation together with endothelial and microvascular dysfunction estimated by the flow-mediated skin fluorescence (FMSF) technique (Table 2). FMSF is one of the available techniques for assessing the function of microcirculation, tissue oxygenation and nutrient supply. This technique is based on the registration of the cutaneous fluorescence intensity of NADH [21]. Excitation of the forearm with ultraviolet light at 340 nm results in the emission of a NADH fluorescence signal from human keratinocytes, which is detected by the receiver diode at 460 nm. The test involves inducing NADH fluorescence during 3 min of brachial artery occlusion [21]. In this way, the ischemic (IR) and hyperemic (HR) responses are recorded. IR reflects tissue sensitivity to hypoxia and includes parameters, such as IR max and IR index. The IR max indicates the ratio of the relative to maximal baseline increase in NADH fluorescence intensity observed over the occlusion period, while the IR index corresponds to the area under the curve. In our previous work [18], as well as in the group of patients recruited in this study, we showed lower IR max and IR index parameters in long COVID compared to healthy controls (Table 2). HR reflects microvascular reactivity and it is quantitatively described by two parameters: HR max and HR index. The first is expressed as the relative to maximal baseline decrease in NADH fluorescence intensity during the reperfusion phase, while the latter is defined as the area under the curve. Although long COVID patients revealed rather minor changes in HR parameters compared to the control group (Table 2), they had lower RHR (reactive hyperemia response), which characterizes endothelial function related predominantly to the production of nitric oxide (NO) in the vasculature due to reactive hyperemia [22]. In addition, long COVID patients revealed a decrease in serum glycine concentration (Table 2), which has been demonstrated as a mitigator of cytokine storm with anti-inflammatory properties in COVID-19 [20]. There were no changes in red and white blood cell parameters in peripheral blood cell count. Meanwhile, differences were noticed in platelet distribution width (PDW) and the percentage of large platelets in favor of these higher parameters in patients after COVID-19 (Table 2).

### 3.2. Adenine Nucleotides’ Concentration in the Erythrocytes of Long COVID Patients Is at Similar Levels as in the Healthy Controls

**Figure 1:**
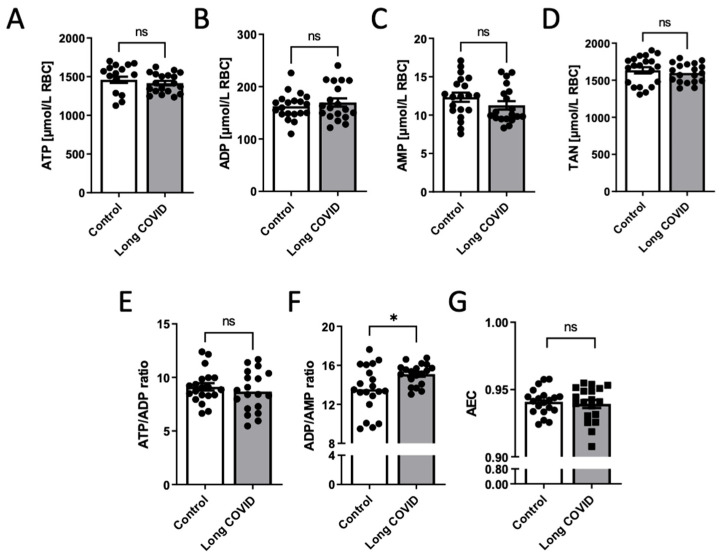
Adenine nucleotides’ concentration in the erythrocytes of long COVID patients is not different from that in healthy controls. The concentration of (**A**) adenosine triphosphate (ATP), (**B**) adenosine diphosphate (ADP), (**C**) adenosine monophosphate (AMP), (**D**) total adenine nucleotide (TAN), (**E**,**F**) adenine nucleotide ratios (ATP/ADP; ADP/AMP) and (**G**) adenylate energy charge (AEC) in red blood cells of post-COVID-19 participants (*n* = 19) compared with healthy control group (*n* = 20). Results are shown as mean ± SEM; * *p* < 0.05 by unpaired Student’s *t*-test (**A**,**B**,**D**–**G**) or Mann–Whitney test (**C**), ns—not significant.

As erythrocytes play a critical role in sufficient tissue oxygenation as well as possibly regulating endothelial and microvascular functions via adenine nucleotide metabolism and signaling, we analyzed the adenine nucleotides in RBCs. The concentrations of ATP, ADP and AMP in the erythrocytes of patients with long COVID did not differ from those in healthy controls (Figure 1). Similarly, no significant differences were observed in the ATP/ADP ratio and adenylate energy charge (AEC). However, the ADP/AMP ratio was higher in long COVID patients.

### 3.3. Adenosine Triphosphate (ATP) Concentration in the Erythrocytes of Long COVID Patients Correlates with Markers of Endothelial and Microcirculatory Function

**Figure 2:**
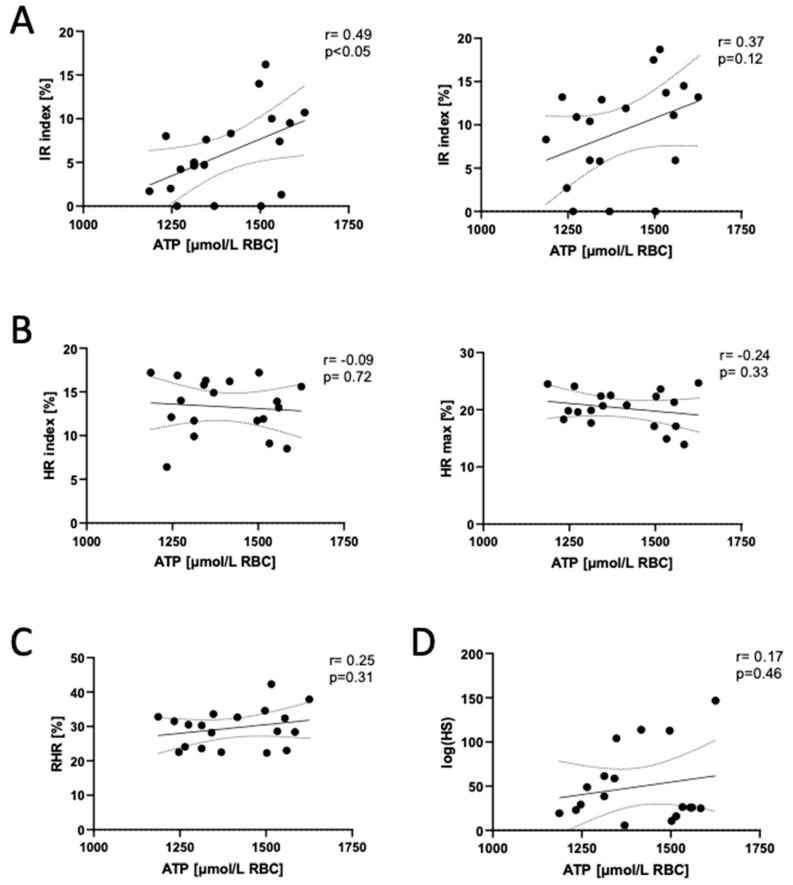
Adenosine triphosphate (ATP) concentration in the erythrocytes of long COVID patients correlates with ischemic response parameters measured by flow-mediated skin fluorescence (FMSF) technique. Correlations of red blood cell adenosine triphosphate (ATP) concentration with (**A**) ischemic response parameters (IR index; IR max), (**B**) hyperemic response parameters (HR index; HR max), (**C**) reactive hyperemic response (RHR) and (**D**) hypoxia sensitivity (logHS) in post-COVID-19 participants. Results are shown as correlation plots with corresponding Pearson (**A**–**C**) or Spearman (**D**) coefficient (r) and *p* value (*p*). Solid line—regression line, dotted line—error bars.

Despite the lack of differences in red blood cell ATP and ADP concentrations between post-COVID-19 patients and healthy controls, we demonstrated a positive correlation of IR max and IR index parameters with erythrocyte ATP concentration (Figure 2) and a negative correlation with ADP (Table S2). This resulted in a strong positive correlation between the IR index, IR max and red blood cell ATP/ADP ratio and AEC (Table S3). In addition, the RHR parameter that reflects the endothelial ability to produce NO negatively correlated with RBC ADP concentration (Table S2) and positively correlated with the RBC ATP/ADP ratio and AEC (Table S3). We also found a negative correlation between log(HS), the other parameter of the microcirculatory response to hypoxia, and the RBC ADP/AMP ratio (Table S3). Log(HS) reflects myogenic microcirculatory oscillations, which are stimulated on the reperfusion line following transient hypoxia [21].

**Figure 3:**
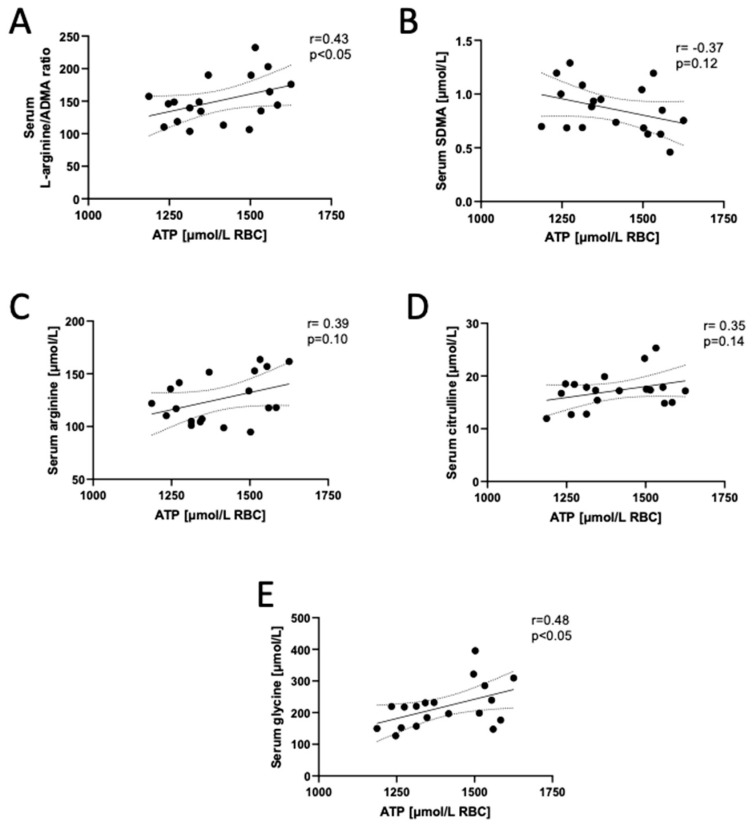
Adenosine triphosphate (ATP) concentration in the erythrocytes of long COVID patients correlates with circulating endothelial function parameters. Correlations of red blood cell adenosine triphosphate (ATP) concentration with (**A**) L-arginine/ADMA (asymmetric dimethyl-L-arginine) ratio, (**B**) symmetric dimethyl L-arginine (SDMA), (**C**) arginine, (**D**) citrulline and (**E**) glycine concentration in long COVID participants. Results are shown as correlation plots with corresponding Pearson coefficient (r) and p value (p). Solid line—regression line, dotted line—error bars.

Additionally, we determined a significant positive correlation between ATP concentration in RBCs and serum arginine/ADMA (a ratio of nitric oxide substrate to NO synthase inhibitor). Then, we found positive relationships between RBC ATP and serum arginine and citrulline concentrations, which are a substrate and co-product in the reaction of NO synthesis, as well as a negative trend between RBC ATP and SDMA (arginine transport inhibitor) (Figure 3). In addition, ATP and TAN concentration in erythrocytes positively correlated with serum glycine concentration, an amino acid with anti-inflammatory properties (Figure 3, Table S2) [23].

### 3.4. Adenosine Triphosphate (ATP) Concentration in the Erythrocytes of Long COVID Patients Correlates with Markers of Systemic Inflammation Reactivation

**Figure 4:**
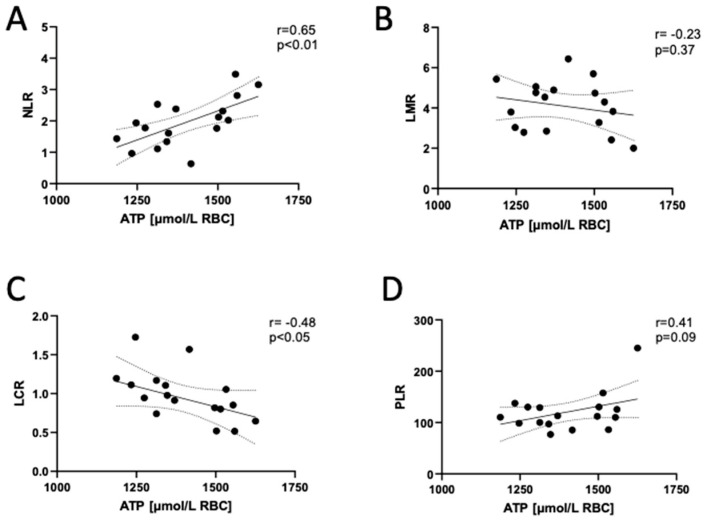
Adenosine triphosphate (ATP) concentration in the erythrocytes of long COVID patients correlates with inflammatory hematological ratios. Correlations of red blood cell adenosine triphosphate (ATP) concentration with (**A**) peripheral blood neutrophil-to-lymphocyte ratio (NLR), (**B**) lymphocyte-to-monocyte ratio (LMR), (**C**) lymphocyte-to-C-reactive protein ratio (LCR) and (**D**) platelet-to-lymphocyte ratio (PLR) in post-COVID-19 participants. Results are shown as correlation plots with corresponding Pearson (**A**–**C**) or Spearman (**D**) correlation coefficient (r) and *p* value (*p*). Solid line—regression line, dotted line—error bars.

Then, we determined no significant correlations of erythrocyte adenine nucleotide concentrations, ATP/ADP or ADP/AMP ratios and AEC with red blood cell parameters (Table 3 and Table S4). However, there was a tendency toward negative relationships between the ATP concentration in erythrocytes and the number of red blood cells, the hematocrit, hemoglobin concentration or red blood cell distribution width (RDW). Interestingly, these trends became weaker or completely disappeared in the correlation analysis with ADP and AMP. Additionally, there were significant relationships between erythrocyte ATP, total adenine nucleotide (TAN) concentration and the percentage of neutrophils (NEU) and lymphocytes (LYMPH) (Table 3), which translated into a strong positive correlation with the neutrophil-to-lymphocyte ratio (NLR) in long COVID participants (Figure 4).

It was observed very soon after the beginning of the COVID-19 pandemic that a high NLR can be used as a reliable indicator to determine disease severity, with a cut-off point above 3.0 [24]. In addition, the NLR has been proposed as a marker of systemic inflammation reactivation when monitoring long COVID patients [25]. It was shown that after normalization to approximately 2.5, the NLR gradually re-elevated to about 3.5 in patients with sustained long COVID symptoms. Our findings indicate that the NLR as well as the lymphocyte-to-C-reactive protein ratio (LCR) significantly correlated with the ATP and TAN concentration in erythrocytes (Figure 3, Table S4). Other indicators, such as the lymphocyte-to-monocyte ratio (LMR) and platelet-to-lymphocyte ratio (PLR), tended to correlate with ATP and TAN concentration in erythrocytes.

## 4. Discussion

This work highlights the potential role of red blood cell adenylate energetics in tissue oxygenation as well as endothelial and microvascular function in patients with long COVID. We demonstrated the correlation of many parameters related to the immune response and endothelial, and microcirculatory function with erythrocyte ATP and TAN concentrations in long COVID patients. A particularly strong positive relationship was observed between ATP concentration in RBCs and the serum ratio of arginine to asymmetric dimethylarginine, an indicator of vascular nitric oxide production capacity. Consistently, a positive correlation was observed between the ATP/ADP ratio in RBCs and diminished reactive hyperemic response in post-COVID-19 patients, assessed by flow-mediated skin fluorescence (FMSF), which reflected decreased vascular NO bioavailability. On the other hand, we have shown that patients with long COVID symptoms have significantly impaired ischemic response parameters (IR max and IR index), examined by FMSF, which revealed diminished residual bioavailability of oxygen in epidermal keratinocytes after brachial artery occlusion. IR max and IR index parameters revealed a strong correlation with the ATP/ADP ratio in RBCs. Taken together, this study indicates that a decrease in peripheral tissue oxygenation in long COVID patients may be associated with diminished intracellular oxygen delivery through the circulatory system due to dysregulation of metabolic processes in erythrocytes, with simultaneous endothelial and microvascular dysfunction. A lack of evident differences in the concentration of adenine nucleotides in the erythrocytes of all post-COVID-19 patients compared to healthy controls may indicate heterogeneity of post-COVID-19 patients, of which a large proportion had restored metabolic equilibrium.

The maintenance of metabolic balance relies significantly on the evolved mechanisms through which hemoglobin in RBCs senses the need for oxygen and responds suitably [26]. The coordinated regulation of ATP production and antioxidant systems within RBCs also takes advantage of Hgb-based oxygen sensitivity to address various physiological and pathological stresses [11]. For instance, during oxygen offloading, glycolysis is promoted to generate both 2,3-DPG (2,3-diphosphoglycerate, a negative allosteric effector of hemoglobin–oxygen binding) and ATP [27]. Conversely, under oxygen-rich conditions, the production via the PPP of nicotinamide adenine dinucleotide phosphate (NADPH), crucial for reducing systems, is favored [27]. The dynamic control of ATP not only ensures the maintenance of the ionic and structural balance in RBCs but also contributes to the availability of vasoregulatory ATP that can be released in hypoxia or during RBC deformation in microvessels [28]. The export of ATP from erythrocytes in response to hypoxia or deformation serves to dilate blood vessels, facilitating efficient oxygen delivery [29].

It has been demonstrated that the adaptability of RBCs to the metabolic environment through the control of the above mechanisms is compromised during COVID-19 infection [27]. It was revealed that RBCs from severe COVID-19 patients displayed signatures of oxidation and fragmentation of key structural and functional proteins including band 3 (AE1), spectrin beta and ankyrin, as well as revealing increased glycolytic intermediates, including 2,3-DPG, without significant changes in ATP levels [16]. Elevations in glycolytic metabolites within RBCs align with the potential enhancement of the capacity of Hgb to offload oxygen as a function of allosteric modulation by high-energy phosphate compounds (a right shift of the oxygen dissociation curve) and may counteract COVID-19-induced hypoxia [30]. However, in spite of high 2,3-DPG levels in COVID-19 patients, no change in hemoglobin affinity was detected in three independent investigations, while in a large cohort study, even a left shift of the oxygen dissociation curve was calculated [4]. The most likely reason for this finding is the formation of methemoglobin, which enhances oxygen affinity and seems to thus counteract the impact of 2,3-DPG. A lack of this back shift would further impede oxygen loading in the damaged lung. The problem is thus partly transferred from oxygen uptake in the lung to oxygen transport from capillaries to the cells that consume it.

In our study, we have shown that patients with long COVID have significantly impaired ischemic response parameters (IR max and IR index), which were examined using the non-invasive FMSF technique. The IR parameters indicate the response to brachial artery occlusion, resulting in the complete blockage of oxygen delivery to the epidermis, and should be treated as a metabolic indicator of the changes in the NADH/NAD^+^ equilibrium in keratinocytes due to transient ischemia [31]. Thus, the gradual shift of the NADH/NAD^+^ equilibrium toward reduction, seen as an increase in NADH fluorescence (ischemic response), depends on the residual bioavailability of oxygen in epidermal keratinocytes after brachial artery occlusion. Therefore, the outcomes of our study confirmed that decreased oxygenation of peripheral tissues may be associated with diminished intracellular oxygen delivery through the circulatory system due to dysregulation of metabolic processes in erythrocytes. This may partly explain long COVID-specific symptoms such as physical impairment and fatigue. In line with that, we have found a decreased log(HS) parameter in post-COVID-19 patients, which mirrors a disturbed microcirculatory response to hypoxia. In another study, low HS values were related to a more severe course of COVID-19, suggesting that this parameter is a prognostic factor of the disease [32]. Interestingly, in this study, log(HS) negatively correlated with the RBC ADP/AMP ratio.

In addition, we revealed a decreased RHR parameter in long COVID patients, reflecting reduced nitric oxide production in the vasculature [22]. This observation is also in line with some other studies [18,33]. Disturbed tissue perfusion may result from both impaired oxygen transport by erythrocytes and dysfunction of the microvascular endothelium. Mounting evidence links SARS-CoV-2 infection with endothelial dysfunction, which has been recognized by reduced nitric oxide bioavailability, oxidative stress, leukocyte adhesion, hyperpermeability, glycocalyx disruption, endothelial-to-mesenchymal transition, hypercoagulability and thrombosis [34]. However, it has been described that COVID-19-induced endotheliitis is predominately a systemic microvessel vasculitis not involving the large arteries such as the main coronaries [35]. In our long COVID patients with typical chest pain and the evidence of ischemia in non-invasive tests, no significant changes were revealed by invasive coronary angiography or cardiac computed tomography angiography. We defined it as a microvascular angina-like phenomenon. In addition, cardiac troponin levels were within reference ranges, as we did not observe any patients during acute coronary syndrome. On the other hand, it should be noted that microvascular and endothelial dysfunction can manifest the autonomic dysfunction in long COVID syndrome, with local symptoms such as headache, brain fog, chest pain, the microvascular angina-like phenomenon, dyspnea and peripheral circulatory symptoms, including skin discoloration, oedema or Raynaud-like phenomena [36]. It is well documented that cardiovascular autonomic dysfunction occurs from a malfunction of the autonomic control of the circulation, and can involve failure or inadequate or excessive activation of the sympathetic and parasympathetic components of the autonomic nervous system [36].

It should be emphasized that beyond the fundamental role of erythrocytes in oxygen transport, RBCs are also critical modulators of endothelial and microvascular function via controlled ATP release [37]. When the mechanisms of ATP synthesis and release function properly, ATP exported from RBCs subserves efficient blood flow, including vasodilation in proportion to the degree of hypoxia, the inhibition of intercellular adhesion and the prevention of unwanted capillary permeability [38]. In our study, low RHR, reflecting lower vascular NO production in long COVID patients, was associated with an increase in ADP concentration in RBCs and a decrease in the ATP/ADP ratio. This is consistent with reports on the stimulation of the first ATP-consuming glycolytic reactions in COVID-19 RBCs. However, it is not known whether this translates into the deregulated release of ATP from erythrocytes. It has been shown that RBC-induced NO-associated vasodilation under hypoxic conditions is the effect of ATP release from RBCs and its interaction via purinergic receptors to stimulate the synthesis of NO by endothelial NO synthase (eNOS) [39]. However, our study shows a positive relationship between the decrease in the serum arginine/ADMA ratio in long COVID patients and the ATP concentration in erythrocytes. This suggests disturbances in ATP release and/or its purinergic signaling cascade. Indeed, recent studies have revealed that long COVID RBCs demonstrated a damaged cell membrane, in particular through the oxidation of band 3 and binding with S1 spike proteins [16,40]. These alterations to band 3 can lead to significant disturbances in RBC functions, including the ATP release mechanism [41]. In such cases, hypoxia seen in COVID-19 patients is related to SARS-CoV-2-mediated band 3 alterations, which may decrease the ability of RBCs to release ATP, reducing vasodilation and oxygen delivery to tissues.

## 5. Conclusions

In this study, we revealed that many parameters related to the endothelial and microvascular function showed significant correlations with red blood cell adenine nucleotide concentration in patients with long COVID. A particularly strong relationship was observed between adenosine triphosphate concentration in erythrocytes and the serum ratio of arginine to asymmetric dimethylarginine—an indicator of endothelial function. In line with that, a positive correlation was observed between the ATP/ADP ratio and diminished reactive hyperemic response in long COVID patients, assessed by the flow-mediated skin fluorescence (FMSF) technique, which reflected decreased vascular nitric oxide bioavailability. In addition, we have shown that patients with long COVID have significantly impaired ischemic response parameters, examined by FMSF, which revealed diminished residual bioavailability of oxygen in epidermal keratinocytes after brachial artery occlusion. Taken together, this study indicates that the dysregulation of metabolic processes in erythrocytes that coexists with endothelial and microcirculatory dysfunction is associated with diminished intracellular oxygen delivery, which can explain long COVID cardiovascular complications, physical impairment and fatigue. In addition, functional assessment of the degree of skin tissue oxygenation using FMSF turned out to be a sensitive method to track the changes in hypoxia occurring after COVID-19. Further studies using this methodology, as well as large-scale analyses of erythrocyte energy metabolism in a larger group of patients, should be also performed.

## Figures and Tables

**Table 1:** General characteristics of long COVID participants compared with the healthy control group. Results are shown as mean ± SEM. Na—not applicable.

Parameter	Control (*n* = 20)	Long COVID (*n* = 19)
Age (years)	40 ± 3	38 ± 2
Sex (F/M)	14/6	12/7
BMI (kg/m^2^)	23.3 ± 0.9	23.5 ± 0.8
Long COVID symptoms		
Fatigue	Na	16 (84%)
Tachycardia	Na	3 (16%)
Chest pain	Na	6 (32%)
Dyspnea	Na	1 (5%)
Headache	Na	1 (5%)
Comorbidities		
Hypothyroidism	5 (25%)	5 (26%)
Asthma	0 (0%)	1 (5%)
Atopic dermatitis	0 (0%)	1 (5%)
Irritable bowel syndrome	1 (5%)	1 (5%)
Depression	0 (0%)	1 (5%)
Hyperlipidemia	0 (0%)	1 (5%)
Diabetes	0 (0%)	1 (5%)
Thrombosis	0 (0%)	1 5%)
Drugs taken		
Levothyroxine	5 (25%)	5 (26%)
Antidepressants	0 (0%)	2 (11%)
Metformin	0 (0%)	1 (5%)
Acetylsalicylic acid	0 (0%)	1 (5%)
Drugs prescribed		
Beta-adrenolytics	0 (0%)	6 (32%)
ACE-inhibitors	0 (0%)	3 (16%)
Statins	0 (0%)	1 (5%)

**Table 2:** Microcirculation function parameters were assessed by flow-mediated skin fluorescence (FMSF) technique, serum circulating inflammatory and endothelial function parameters, and peripheral blood cell count in long COVID participants compared with healthy control group. Results are shown as mean ± SEM with corresponding *p*-value by unpaired Student’s *t*-test or Mann–Whitney test as appropriate. IR—ischemic response, HR—hyperemic response, RHR—reactive hyperemic response, log(HS)—hypoxia sensitivity parameter, hs-CRP—high-sensitive C-reactive protein, RBCs—red blood cells, Hct—hematocrit, Hgb—hemoglobin, MCV—mean corpuscular volume, MCH—mean corpuscular hemoglobin, MCHC—mean corpuscular hemoglobin concentration, RDW—red blood cell distribution width, WBC—white blood cells, NEU—neutrophils, LYMPH—lymphocytes, MONO—monocytes, EOS—eosinophils, BAS—basophils, PLT—platelets, PDW—platelet distribution width, PCT—plateletcrit, NLR—neutrophil-to-lymphocyte ratio, LMR—lymphocyte-to-monocyte ratio, LCR—lymphocyte-to-C-reactive protein ratio and PLR—platelet-to-lymphocyte ratio.

Parameter	Control	Long COVID	*p* Value
Microcirculatory function parameters			
IR index [%]	14.5 ± 1.74	6.06 ± 1.09	<0.001
IR max [%]	19.5 ± 2.12	9.30 ± 1.32	<0.001
HR index [%]	15.1 ± 1.15	13.3 ± 0.73	0.20
HR max [%]	22.3 ± 2.29	20.3 ± 0.72	0.42
RHR [%]	38.7 ± 2.24	30.8 ± 1.32	<0.01
Log (HS)	114 ± 38.7	49.6 ± 9.85	<0.05
Serum inflammatory parameters			
hs-CRP [mg/L]	2.1 ± 0.30	2.4 ± 0.31	0.489
Serum amino acid compounds			
Arginine [μmol/L]	112 ± 6.92	126 ± 5.27	0.12
Citrulline [μmol/L]	15.5 ± 0.93	17.2 ± 0.76	0.17
SDMA [μmol/L]	0.83 ± 0.04	0.86 ± 0.05	0.64
Arginine/ADMA	162 ± 8.00	151 ± 8.08	0.34
Glycine [μmol/L]	314 ± 37.4	219 ± 12.9	<0.05
Peripheral blood cell count			
RBCs [T/L]	4.86 ± 0.10	4.70 ± 0.09	0.26
Hct [%]	42.4 ± 0.74	42.2 ± 0.75	0.25
Hgb [g/dL]	14.3 ± 0.29	14.2 ± 0.29	0.79
MCV [fL]	88.7 ± 0.91	87.7 ± 0.95	0.45
MCH [pg]	30.5 ± 0.32	30.2 ± 0.46	0.60
MCHC [g/dL]	34.3 ± 0.32	34.4 ± 0.31	0.81
RDW [%]	12.9 ± 0.18	12.8 ± 0.20	0.85
WBC [G/L]	6.22 ± 1.81	6.28 ± 0.47	0.96
NEU [G/L]	3.52 ± 1.22	3.65 ± 0.37	0.92
NEU [%]	56.6 ± 2.11	56.5 ± 2.21	0.98
LYMPH [G/L]	2.31 ± 0.23	1.94 ± 0.11	0.16
LYMPH [%]	37.1 ± 2.43	32.3 ± 2.03	0.14
MONO [G/L]	0.48 ± 0.05	0.51 ± 0.04	0.64
MONO [%]	7.72 ± 0.19	8.22 ± 0.48	0.33
EOS [G/L]	0.12 ± 0.03	0.15 ± 0.02	0.42
EOS [%]	1.93 ± 0.21	2.35 ± 0.26	0.21
BAS [G/L]	0.04 ± 0.01	0.04 ± 0.01	0.99
BAS [%]	0.64 ± 0.06	0.56 ± 0.05	0.31
PLT [G/L]	216 ± 12.0	222 ± 11.0	0.72
PDW [fL]	10.9 ± 0.51	13.2 ± 0.41	<0.01
PCT [%]	0.23 ± 0.01	0.24 ± 0.01	0.48
Large PLT [%]	26.7 ± 2.31	32.3 ± 1.44	<0.05
NLR	1.52 ± 0.12	1.93 ± 0.18	0.06
LMR	4.81 ± 0.32	4.11 ± 0.29	0.11
LCR	1.10 ± 0.09	0.94 ± 0.08	0.19
PLR	93.5 ± 10.4	120 ± 8.80	0.06

**Table 3:** Adenosine triphosphate (ATP) and total adenine nucleotide (TAN) concentration in the erythrocytes of long COVID patients correlates with peripheral blood neutrophil and lymphocyte percentage. Correlations of red blood cell adenosine triphosphate (ATP), adenosine diphosphate (ADP), adenosine monophosphate (AMP) and total adenine nucleotide (TAN) concentration with peripheral blood cell count in long COVID participants (*n* = 19). Results are shown as Pearson or Spearman (as appropriate) correlation coefficient (r) and *p* value (*p*).

Parameter	ATP [μmol/L RBC]		ADP [μmol/L RBC]		AMP [μmol/L RBC]		TAN [μmol/L RBC]	
	r	*p* Value	r	*p* Value	r	*p* Value	r	*p* Value
RBC [T/L]	−0.28	0.26	0.12	0.63	0.08	0.75	−0.23	0.36
Hct [%]	−0.27	0.28	0.14	0.57	0.04	0.87	−0.21	0.39
Hgb [g/dL]	−0.21	0.42	0.13	0.61	0.07	0.79	−0.16	0.53
MCV [fL]	0.08	0.74	0.04	0.87	−0.04	0.87	0.09	0.73
MCH [pg]	0.23	0.37	0.18	0.47	0.03	0.90	0.23	0.37
MCHC [g/dL]	0.14	0.59	−0.01	0.99	0.07	0.77	0.13	0.61
RDW [%]	−0.34	0.17	−0.17	0.50	−0.17	0.49	−0.37	0.13
WBC [G/L]	0.26	0.30	−0.10	0.70	−0.16	0.54	0.22	0.37
NEU [G/L]	0.39	0.11	−0.03	0.91	−0.12	0.64	0.37	0.13
NEU [%]	0.55	<0.05	0.07	0.78	−0.06	0.80	0.55	<0.05
LYMPH [G/L]	−0.24	0.34	−0.17	0.50	−0.11	0.66	−0.27	0.27
LYMPH [%]	−0.52	<0.05	0.01	0.97	−0.13	0.60	−0.50	<0.05
MONO [G/L]	0.01	0.98	−0.42	0.08	−0.40	0.10	−0.11	0.66
MONO [%]	−0.28	0.26	−0.40	0.10	−0.32	0.20	−0.38	0.12
EOS [G/L]	0.12	0.63	0.06	0.83	0.02	0.95	0.13	0.60
EOS [%]	−0.04	0.89	0.07	0.78	0.09	0.71	−0.02	0.95
BAS [G/L]	0.01	0.97	−0.15	0.54	−0.14	0.59	−0.04	0.89
BAS [%]	−0.27	0.28	−0.02	0.94	0.05	0.84	−0.27	0.28
PLT [G/L]	0.07	0.79	0.08	0.75	0.03	0.92	0.09	0.73
PDW [fL]	0.33	0.18	−0.21	0.40	−0.28	0.26	0.26	0.30
PCT [%]	0.18	0.48	0.01	0.97	−0.06	0.80	0.17	0.49
Large PLT [%]	0.32	0.20	−0.25	0.31	−0.32	0.20	0.24	0.35

## Data Availability

The data presented in this study are available on request from the corresponding author.

## Supplementary Materials

The following supporting information can be downloaded at: https://www.mdpi.com/article/10.3390/biomedicines12030554/s1, Table S1. Dates of COVID-19 diagnosis, peripheral blood sample collection and FMSF testing in recruited patients. F—female. M—Male. Table S2. Correlations of red blood cell adenosine diphosphate (ADP), adenosine monophosphate (AMP) and total adenine nucleotide (TAN) concentration with L-arginine/ADMA (asymmetric dimethyl-L-arginine) ratio and symmetric dimethyl L-arginine (SDMA) concentration in long COVID participants. Results are shown as Pearson correlation coefficient (r) and *p* value (*p*). Table S3. Correlations of red blood cell adenine nucleotide ratios and adenylate energy charge (AEC) with peripheral blood cell count in long COVID participants. Results are shown as Pearson correlation coefficient (r) and *p* value (*p*). Table S4. Correlations of red blood cell adenine nucleotide ratios and adenylate energy charge (AEC) with peripheral blood cell count in long COVID participants. Results are shown as Pearson correlation coefficient (r) and *p* value (*p*). Table S5. Correlations of red blood cell adenosine diphosphate (ADP), adenosine monophosphate (AMP) and total adenine nucleotide (TAN) concentration with peripheral blood cell count in long COVID participants. Results are shown as Pearson correlation coefficient (r) and *p* value (*p*).
