# Supplementary material for: Red Blood Cell Adenylate Energetics Is Related to Endothelial and Microvascular Function in Long COVID
Source: Biomedicines. 2024 Mar 1;12(3):554. doi: 10.3390/biomedicines12030554 (PMC10968064; doi:10.3390/biomedicines12030554)
Supplement: Supplementary file 1 [file biomedicines-12-00554-s001.zip › biomedicines-2878488-supplementary.pdf]

Supplementary material

Red Blood Cell Adenylate Energetics is Related to Endothelial and Microvascular Function in Long COVID

Patient	Age (years)	Gender	COVID19 diagnosis	Blood sample collection/ FMSF testing	Time interval (days)	Post-COVID19 symptoms
1	43	F	22/12/2020	15/02/2021	55	tachycardia, fatigue
2	37	F	08/01/2021	22/02/2021	45	dyspnea, chest pain
3	50	F	13/12/2020	22/02/2021	71	fatigue, headache
4	45	F	01/11/2020	22/02/2021	113	fatigue
5	48	F	25/11/2020	22/02/2021	89	fatigue, chest pain
6	43	F	25/11/2020	01/03/2021	96	chest pain
7	34	F	26/10/2020	01/03/2021	126	fatigue
8	47	F	01/11/2020	01/03/2021	120	fatigue
9	44	F	07/12/2020	15/03/2021	98	fatigue
10	25	F	15/12/2020	15/03/2021	90	chest pain
11	23	F	26/11/2020	15/03/2021	109	fatigue
12	27	F	14/12/2020	15/03/2021	91	tachycardia, fatigue
13	38	M	24/02/2021	15/03/2021	19	fatigue, chest pain
14	45	M	26/11/2020	22/03/2021	116	fatigue
15	23	M	01/01/2021	15/02/2021	45	tachycardia, fatigue
16	45	M	21/10/2020	22/02/2021	124	fatigue
17	25	M	01/11/2020	22/02/2021	113	fatigue
18	25	M	08/12/2020	01/03/2021	83	fatigue, dyspnea
19	48	M	01/12/2020	01/03/2021	90	fatigue, chest pain

Table S1. Dates of COVID19 diagnosis, peripheral blood sample collection, and FMSF testing in recruited patients. F – female, M – Male.

Parameter	ADP [µmol/L RBC]		AMP [µmol/L RBC]		TAN [µmol/L RBC]	
	r	p value	r	p value	r	p value
Arginine [µmol/L]	-0.30	0.22	-0.27	0.26	0.30	0.21
Citrulline [µmol/L]	-0.16	0.51	-0.01	0.96	0.30	0.21
SDMA [µmol/L]	-0.09	0.72	0.03	0.89	-0.38	0.11
Arginine/ADMA	0.06	0.81	-0.11	0.65	0.42	0.08
Glycine [µmol/L]	0.05	0.81	0.13	0.60	0.49	<0.05
IR index [%]	-0.46	<0.05	-0.34	0.15	0.36	0.14
IR max [%]	-0.59	<0.01	-0.47	<0.05	0.21	0.40
HR index [%]	0.25	0.31	0.22	0.37	0.03	0.91
HR max [%]	0.20	0.41	0.10	0.68	-0.18	0.45
RHR [%]	-0.49	0.03	-0.42	0.07	0.11	0.66
Log (HS)	-0.05	0.85	0.13	0.60	-0.04	0.88

Table S2. Correlations of red blood cell adenosine diphosphate (ADP), adenosine monophosphate (AMP), and total adenine nucleotide (TAN) concentration with L-arginine/ADMA (asymmetric dimethyl-L-arginine) ratio, symmetric dimethyl L-arginine (SDMA) concentration in long COVID19 participants. Results are shown as Pearson correlation coefficient (r) and p value (p).

Parameter	RBC ATP/ADP		RBC ADP/AMP		RBC AEC	
	r	p value	r	p value	r	p value
Arginine [μmol/L]	0.42	0.08	-0.09	0.71	0.40	0.09
Citrulline [μmol/L]	0.37	0.13	-0.52	<0.05	0.25	0.30
SDMA [μmol/L]	-0.03	0.89	-0.42	0.07	-0.10	0.67
Arginine/ADMA	0.05	0.83	0.49	<0.05	0.17	0.49
Glycine [μmol/L]	0.20	0.41	-0.28	0.25	0.14	0.58
IR index [%]	0.65	<0.01	-0.33	0.17	0.56	<0.05
IR max [%]	0.68	<0.01	-0.30	0.21	0.64	<0.05
HR index [%]	-0.26	0.26	0.14	0.56	-0.20	0.41
HR max [%]	-0.34	0.15	0.35	0.14	-0.23	0.35
RHR [%]	0.51	<0.01	-0.11	0.65	0.52	<0.05
Log (HS)	0.05	0.85	-0.48	<0.05	0.01	0.99

Table S3. Correlations of red blood cell adenine nucleotide ratios and adenylate energy charge (AEC) with peripheral blood cell count in long COVID19 participants. Results are shown as Pearson correlation coefficient (r) and p value (p).

Parameter	RBC ATP/ADP		RBC ADP/AMP		RBC AEC	
	r	p value	r	p value	r	p value
RBC [T/L]	-0.21	0.41	0.08	0.75	-0.29	0.25
Hct [%]	-0.25	0.33	0.26	0.30	-0.29	0.25
Hgb [g/dL]	-0.23	0.37	0.18	0.48	-0.24	0.34
MCV [fL]	-0.06	0.70	0.29	0.24	0.04	0.88
MCH [pg]	-0.04	0.88	0.16	0.55	0.09	0.74
MCHC [g/dL]	0.02	0.92	-0.16	0.54	0.07	0.77
RDW [%]	0.04	0.86	-0.03	0.89	0.03	0.92
WBC [G/L]	0.21	0.40	0.12	0.63	0.18	0.48
NEU [G/L]	0.21	0.40	0.19	0.45	0.16	0.52
NEU [%]	0.20	0.43	0.27	0.28	0.13	0.61
LIMF [G/L]	0.07	0.78	-0.14	0.59	0.07	0.79
LIMF [%]	-0.24	0.33	-0.25	0.31	-0.19	0.44
MONO [G/L]	0.36	0.14	-0.01	0.96	0.38	0.12
MONO [%]	0.19	0.45	-0.15	0.55	0.26	0.29
EOS [G/L]	-0.02	0.95	0.11	0.65	-0.01	0.99
EOS [%]	-0.11	0.95	-0.01	0.98	-0.08	0.75
BAS [G/L]	0.08	0.66	-0.01	0.97	0.17	0.50
BAS [%]	-0.18	0.74	-0.12	0.64	-0.06	0.83
PLT [G/L]	-0.10	0.71	0.15	0.56	-0.05	0.83
PDW [fL]	0.37	0.14	0.17	0.51	0.34	0.17
PCT [%]	0.02	0.93	0.19	0.44	0.06	0.82
Large PLT [%]	0.39	0.11	0.17	0.49	0.37	0.13
NLR	0.21	0.41	0.29	0.25	0.16	0.53
LMR	-0.32	0.20	-0.13	0.60	-0.35	0.15
LCR	0.13	0.60	-0.01	0.87	-0.20	0.43
PLR	-0.01	0.97	0.21	0.40	0.02	0.93

Table S4. Correlations of red blood cell adenine nucleotide ratios and adenylate energy charge (AEC) with peripheral blood cell count in long COVID19 participants. Results are shown as Pearson correlation coefficient (r) and p value (p).

Parameter	ADP [μmol/L RBC]		AMP [μmol/L RBC]		TAN [μmol/L RBC]	
	r	p value	r	p value	r	p value
NLR	0.08	0.75	-0.05	0.85	0.65	<0.01
LMR	0.30	0.22	0.35	0.16	-0.13	0.60
LCR	-0.37	0.13	-0.34	0.17	-0.48	<0.05
PLR	0.13	0.59	0.05	0.84	0.42	0.08

Table S5. Correlations of red blood cell adenosine diphosphate (ADP), adenosine monophosphate (AMP), and total adenine nucleotide (TAN) concentration with peripheral blood cell count in long COVID19 participants. Results are shown as Pearson correlation coefficient (r) and p value (p).
